# Smartphone Sensors for Indoor Positioning

**DOI:** 10.3390/s23083811

**Published:** 2023-04-07

**Authors:** Imran Ashraf, Yongwan Park, Yousaf Bin Zikria, Sadia Din

**Affiliations:** 1 Department of Information and Communication Engineering, Yeungnam University, Gyeongsan 38541, Republic of Korea; 2 Victorian Institute of Technology (VIT), 157/161 Gloucester St, The Rocks, Sydney, NSW 2000, Australia; 3 Department of Electrical and Computer Engineering, Texas A&M University, Doha 23874, Qatar

## 1. Introduction

The explosive growth and wide proliferation of mobile devices, the majority of which are smartphones, led to the inception of several novel and intuitive services, including on-the-go services, online customer services, and location-based services (LBS). The industry support services and products for customers based on their current location include product sales, entertainment, and other services such as filling stations in the vicinity, etc. This indicates that precise location information is very important for offering better services and a higher quality of satisfaction for users. However, positioning, especially indoor positioning still faces many challenges. For outdoor environments, a global positioning system (GPS) can provide metre-level accuracy that meets the current requirements of outdoor services for LBS. Unfortunately, the same cannot be said for indoor environments. GPSs have several limitations indoors including, but not limited to, signal absorption, shadowing, signal fading, etc. [1].

Recent technological advancements have led to novel sensing technologies that offer small yet powerful sensors that provide a large variety of data. Several solutions have been proposed to overcome the limitations of GPS and its alternatives, such as wireless local area networks, Bluetooth low energy (BLE), ultra-wideband (UWB), magnetic field positioning, and pedestrian dead reckoning (PDR). Not to mention the advantages offered by 5G communication technologies which has opened new possibilities for cellular-based positioning. However, these technologies come with their own challenges and shortcomings when precise positioning is concerned with the indoor environment. These limitations stem from inherent limitations of the used technology, the influence of the external environment, the dependence on dedicated infrastructure, dynamic agents in the environment, and requirements regarding latency. Moreover, these technologies provide poor accuracy for LBS, when used alone. Multi-sensor solutions have been found to be more accurate [2].

With the excessive use of smartphones by the masses globally, more and more sensors are being embedded in smartphones. Just take the example of the iPhone which now contains a UWB sensor that can be used for centimetre-level positioning. Consequently, the focus has now shifted towards smartphone-based positioning where the built-in sensors are leveraged to locate the user in real-time. However, to do so, novel fusion algorithms, multi-modal data gathering and analysis frameworks, and artificial intelligence-based robust solutions are needed to serve the requirements of LBS. For this purpose, the role of sensors for accurate and noise-tolerant data collection and machine learning techniques has become very important. With the launch of 5G, robust and lower-latency solutions can be obtained for real-time applications.

## 2. Current Special Issue

The current Special Issue on smartphone sensor-based positioning has published nine research articles. These papers have utilized different sensors and approaches for indoor positioning and are predominantly based on machine learning and deep learning solutions. In addition, the pros and cons of the different smartphone sensors are discussed concerning positioning performance. For example, one paper proposes a zero infrastructure-based indoor positioning system for handheld devices. Similarly, a conceptual 6G architecture is presented for future use. The use of smart sensors is elaborated upon in one paper for railway track localization and inspection. One paper presents wireless optical communication for road and pedestrian safety. WiFi access point design is also presented for indoor positioning of the internet of things (IoT) and smartphones. Similarly, the use of smartphone sensors for early diagnosis of coronavirus has been researched in one of the papers. One paper proposes a light positioning framework using an optical frequency division multiplex (OFDM) approach. Finally, the last paper provides an empirical overview of open-source datasets for magnetic field-based indoor positioning. A comparative summary of the published papers is given in Table 1 while the subsequent sections contain detailed discussions.

The authors in [3] present the study of smartphone sensors, algorithms, and techniques to perform indoor localization. The focus is especially placed on user tracking without a dedicated infrastructure or additional hardware. By analysing the strengths and limitations of each component, a handheld device-based indoor localization approach is devised with zero infrastructure. An accelerometer, magnetometer, gyroscope, and the smartphone camera are utilized in the study. The proposed solution is merged with the Web of Things (WoT) through NodeRED. The proposed framework comprises three levels of smoothing, initial fusion, and fusion at the top level. The first level prepares data for processing before it can be used for initial fusion which is performed in the second level where handheld devices are interconnected for machine-to-machine communication. In the third level, the machine learning model is combined with pre-processed data and a camera to guide the user in particular directions indoors.

A conceptual nested bee-hive architecture, a terrestrial network, is presented in [4] for future smart cities. The proposed approach aims to provide device connectivity for future sensors with huge data traffic. It provides ubiquity, a higher QoS, and on-demand content for massive interconnected devices. Based on terahertz (THz) wave propagation, it will provide up to one terabit per second (Tbps). In addition, the multilayer architecture is designed to ensure scalability to adjust for future needs. Extensive simulations are carried out using different pathfinding algorithms, such as 3D-directional greed and 3D-Dijkstra, to optimize the communication path. Performance is evaluated in terms of the cost and number of hops, dynamic network slicing, security, load distribution, IoT support, and backhaul. Results prove the 6G nested bee-hive to be a low-latency, artificial intelligence (AI)-centric, multilayer physical network of high bandwidth for long- and medium-distance communication.

A high-accuracy railway track inspection and localization approach is presented in [5] with a 99.7% accuracy. Keeping in view the importance of railway track inspection and fault localization, highly accurate solutions are needed to avoid serious accidents. Image processing approaches are computational-intensive, thus requiring an expensive setup for monitoring and analysing track conditions. The study, on the other hand, employs audio sensors for data collection and utilizes acoustic analysis for track detection and localization. Deep learning models have proven to be more accurate and robust against noise, and the study adopts the long short-term memory (LSTM) model. The proposed solution is an on-the-fly approach and can provide accurate results in real time. In addition, data augmentation is carried out and experiments are performed using various combinations of data augmentation. The best results are obtained using the LSTM model with 8.5 split times.

A visible light-based solution is built in [6] to provide safe and reliable communications. The proposed solution aims to provide pedestrian safety and improve road traffic safety. The challenges of visible light communication, such as dynamicity and exposure to noise, are thoroughly investigated to develop dedicated applications. Developing applications dedicated to direct communications with infrastructure and vehicles using portable devices is becoming a challenge and at the same time a necessary solution. The shaping of an emission–reception architecture is proposed for adaptive fused light communications. The optical camera communication is used via ambient light sensors in Android. The visible light sources on the roadside are used such as traffic signals, traffic signs, road lights, etc. Vehicle-to-infrastructure communication is obtained using the available light-emitting diode (LED) units alongside the road and a dedicated Android app. The research presents preliminary results where the smartphone camera and ambient light sensor can be used to communicate with road infrastructure, vehicles, and pedestrians.

The study [7] highlights the importance of indoor positioning for diverse applications, especially in the context of smartphones. In addition, the case of IoT concerning the requirements for device-to-device (D2D) communication is considered. For D2D communication, the clients need to have minimal software modules to carry out indoor positioning. To satisfy these requirements, the study presents the use of WiFi technology using two orthogonal-phased antenna arrays. For a fully automatic solution, the designed approach utilizes Raspberry Pi, external WiFi modules, orthogonal-phased antenna arrays, and a universal serial bus (USB) hub. Extra-phase circuits are used with radio frequencies to govern phases of the antenna array. The results are based on proof-of-concept commercial APs which show the efficacy of the proposed WiFi AP design.

Smartphone sensors can be utilized for diverse applications including positioning, health informatics, and disease detection. A similar disease detection framework is formulated in [8] to strengthen the healthcare system. The method aims to reduce the number of physical visits to hospitals and medical tests needed for COVID-19, such as nucleic acid amplification tests, computer tomography, and other blood tests. Micro-electro-mechanical-systems sensors, embedded in modern smartphones, are used to record the physical movements of users, temperature readings, and cough sounds with audio sensors. Such information is enough to perform a preliminary analysis of symptoms related to COVID-19. The proposed framework is easy to use and can be quickly deployed to screen COVID-19 patients. Results show a promising accuracy of 79% based on the smartphone sensors data alone which is quite good considering that no physical visits and blood tests are needed.

Smart sensors have been utilized for timely inspection of railway track detection and localization using the audio data in [9]. Different kinds of faults are detected such as normal track, wheel burnt, and super-elevation. Contrary to manual inspection, which is subject to error and is time-consuming, the proposed approach is automatic, thus requiring no human intervention. Mel-frequency cepstral coefficient (MFCC) features from the acoustic data are used for the proposed approach which proves to be capable of differentiating between different kinds of faults. The random forest (RF) and decision tree (DT) machine learning models prove to be highly accurate providing a 97% accuracy for fault detection.

For visible light communication (VLC), the design of solid-state lighting is investigated in [10]. Keeping in view its importance and usage in intelligent buildings, VLC communication, and IoT, the study determines the power and delay spread from a light source to a receiver plane. Power estimation is carried out using the position of the light source and receiver. In particular, analogue OFDM in VLC and assessing the area under the curve (AUC) is emphasized. For the system design, quadrature amplitude modulation (QAM) and pulse-width modulation (PWM) are used for VLC communication and dimming sources, respectively. For measuring the power of LEDs, the receiver plane is analysed. A proof of concept is provided and results demonstrate a 30% increase in the AUC when changing the number of LEDs from four to three. The system is reported to achieve better energy consumption compared to existing techniques.

The final paper [11] is a state-of-the-art review of the open-source benchmark datasets for magnetic field data to perform smartphone-based indoor positioning. Starting with a brief overview of the Earth’s magnetic field and its properties, important aspects of the magnetic field are discussed. It is followed by the challenges of magnetic field-based positioning, such as device diversity, user diversity, space diversity, and time diversity. In addition, the impact of the dynamic behaviour of external agents in a given environment is also analysed. The internal factors of the device associated with the change in the magnetic field data are also discussed, such as device orientation and heterogeneity of the embedded sensors. Similarly, the impact of walking speed and path trajectory is also investigated.

The study presents six open-source datasets for smartphone magnetic sensor-based indoor positioning. Each dataset is discussed in great detail including its collection process, path geometry, place dimensions, and data collection scenarios. Moreover, the number and details of data collection participants in terms of gender and height are also given. The detailed specification of the smartphones and accompanying embedded sensors is also given. The pros and cons of each dataset are elaborated extensively to guide the reader on the use of particular datasets for particular objectives. In the end, a comprehensive overview of the limitations of existing benchmarks is presented and future directions are laid out.

## 3. Conclusions

The papers published in the Special Issue cover different aspects of indoor positioning and localization and can be used to outline future directions.

First, different studies have investigated the pros and cons of different technologies for smartphone-based indoor positioning, such as a magnetic field, WiFi, PDR, visible light, etc. It has been observed that accuracy can dramatically vary with respect to several elements, such as the choice of positioning algorithm, channel model, dynamicity of the environment, number of RPs used for positioning, and latency requirement. Often, there is a trade-off between latency and positioning accuracy or positioning accuracy and cost.

Second, there is no single solution to precise indoor positioning primarily due to the varying needs of positioning accuracy, the complexity of indoor structures, and latency requirements for real-time environments. Multi-modal solutions are best suited for indoor environments and have proven to be more accurate than a single technology. However, sensor fusion requires complex fusion frameworks and often needs high-computational resources and energy consumption.

Third, machine learning and deep learning have been readily adopted for better solutions for indoor positioning. With the availability of 5G communication, especially D2D communication, positioning accuracy can be greatly increased without introducing much time delay. Furthermore, custom-designed WiFi APs for indoor positioning systems and architecture for 6G communication could greatly help enhance positioning accuracy.

Finally, the research efforts for visible light communication are also on the rise which, although in its infancy, could provide efficient solutions for short-range communication and positioning. The use of UWB sensors in iPhones is also under study for precise indoor positioning. Several novel D2D solutions are also underway. Besides positioning accuracy, aspects related to privacy, data confidentiality, and security also deserved to be investigated.

## Figures and Tables

**Table 1:** Analytical overview of the research works published in the Special Issue.

Ref.	Objective	Data/Tool	Proposed/Adopted Approach
[3]	Infrastructure-free positioning	Accelerometer, gyroscope, magnetometer, camera	Machine learning, smartphone sensors, WoT
[4]	6G communication	Terahertz wave propagation	Nested bee-hive for low-latency, high-bandwidth communication for massive interconnected devices.
[5]	Railway track detection and localization	Audio data	LSTM with data augmentation to offer highly accurate on-the-fly solution.
[6]	Visible light communication	Smartphone camera, ambient light sensor and roadside LEDs	VLC-based solution for road traffic and pedestrian safety
[7]	WiFi AP design	Raspberry Pi, USB, WiFi modules, RF switches	A WiFi AP design to meet the needs of indoor positioning systems.
[8]	Early screening of COVID-19	Smartphone sensors	Initial screening of COVID-19 using temperature, cough sounds, and other symptoms recorded using smartphone sensors.
[9]	Fault detection and localization	MFCC features from acoustic data	Highly accurate railway track fault detection and localization approach.
[10]	Visible light communication	OFDM, QAM, PWM	Better AUC for VLC and better energy consumption.
[11]	Magnetic field-based positioning	Smartphone magnetometer sensor	Analytical review of open source benchmarks.
